# Role of the faecolith in modern-day appendicitis

**DOI:** 10.1308/003588413X13511609954851

**Published:** 2013-01

**Authors:** JP Singh, JG Mariadason

**Affiliations:** Metropolitan Hospital, New York,US

**Keywords:** Appendicitis, Faecoliths, Pathology, Computed tomography

## Abstract

**Introduction:**

The prevailing view on appendicitis is that the main aetiology is obstruction owing to faecoliths in adults and lymphoid hyperplasia in children. Faecoliths on imaging studies are believed to correlate well with appendicitis.

**Methods:**

A retrospective chart review was conducted of 1,014 emergency appendicectomy patients between 2001 and 2011. Faecolith prevalence in adult and paediatric appendicectomy specimens with and without perforation was studied. The sensitivity and positive predictive value (PPV) of computed tomography (CT) for identifying faecoliths in the pathology specimen were examined.

**Results:**

Overall, faecoliths were found in 18.1% (178/986) of appendicitis specimens and 28.6% (8/28) of negative appendicectomies. Faecolith prevalence for positive cases was 29.9% (79/264) in paediatric patients and 13.7% (99/722) in adults (*p*<0.05). Faecolith prevalence was 39.4% in perforated appendicitis but only 14.6% in non-perforated appendicitis (*p*<0.05). In adults, faecolith prevalence was 27.5% in perforated appendicitis and 12.0% in non-perforated appendicitis (*p*<0.05) while in paediatric patients, it was 56.1% in perforated appendicitis and 22.7% in non-perforated appendicitis (*p*=0.00). Sensitivity and PPV of preoperative CT in identifying faecoliths on pathology were 53.1% (86/162) and 44.8% (86/192) respectively.

**Conclusions:**

Faecolith prevalence is too low to consider the faecolith the most common cause of non-perforated appendicitis. Faecoliths are more prevalent in paediatric appendicitis than in adult appendicitis. Preoperative CT is an unreliable predictor of faecoliths in pathology specimens.

Medical textbooks still teach the doctrine that the main cause of appendicitis is obstruction of the lumen of the appendix by lymphoid hyperplasia or faecoliths.^1–4^ The former is considered the main cause in children and the latter in adults although foreign bodies, parasites and tumours have also been implicated.

While earlier reports showed a high prevalence of faecoliths in appendicitis,^5^ they included a large proportion of cases of perforated and gangrenous appendicitis. More recent reports (with lower perforation rates) show a much lower prevalence of faecoliths, especially in non-perforated appendicitis. This study was undertaken to test the validity of the teaching that faecoliths are still the main cause of appendicitis by examining the prevalence of faecoliths in appendicitis at our institution. The accuracy of computed tomography (CT; used liberally at our institution since 2001) in predicting the presence of faecoliths in the appendicectomy specimen was also studied. We believe that this is the first such study.

## Methods

A retrospective chart review of all patients who underwent an appendicectomy at Metropolitan Hospital, New York, between 2001 and 2011 was undertaken after obtaining institutional review board approval as well as approval from New York Medical College. A study on negative appendicectomy rate (NAR) was submitted separately.^6^ The prevalence of faecoliths in the pathology specimens of adults and paediatric patients who underwent an appendicectomy for a diagnosis of appendicitis was studied. The sensitivity and positive predictive value (PPV) of CT in identifying faecoliths in pathology specimens were examined.

Appendicitis was defined as the presence of inflammatory cells in the appendix. Absence of inflammation in the appendix was deemed a negative appendicectomy. Perforation was defined as perforation of the appendix demonstrated on histopathology. Faecoliths (synonyms: appendicoliths, coproliths, stercoliths) were defined as faecal concretions or pellets. Calcified faecoliths and appendiceal calculi were included without separate classification. Paediatric patients were identified as age 17 and under and adults as age 18 and over.

Since 2004, CT has been performed at our institution with a 16-slice GE Healthcare (Little Chalfont, UK) scanner using oral contrast for suspected appendicitis.

The data were analysed with Fisher’s exact test using SPSS^®^ (SPSS, Chicago, IL, US). Odds ratio (OR), positive predictive value (PPV) and *p*-value were calculated where relevant.

**Table 1:** Faecolith prevalence rates in adult and paediatric appendicectomies

Appendicectomies	Overall	Adult (18 yrs +)	Paediatric (≤17 yrs)
All cases	1,014	741	273
Positive on pathology	986/1,014 = 97.2%	722/741 = 97.4%	264/273 = 96.7%
Negative on pathology	28/1,014 = 2.8%	19/741 = 2.6%	9/273 = 3.3%
Faecoliths in positive appendicectomies	178/986 = 18.1%	99/722 = 13.7%	79/264 = 29.9%
Faecoliths in negative appendicectomies	8/28 = 28.6%	6/19 = 31.6%	2/9 = 22.2%
Perforated appendicitis	137/986 = 13.9%	80/722 = 11.1%	57/264 = 21.6%
Faecoliths in perforated appendicitis	54/137 = 39.4%	22/80 = 27.5%	32/57 = 56.1%
Faecoliths in non-perforated appendicitis	124/849 = 14.6%	77/642 = 12.0%	47/207 = 22.7%

## Results

After excluding incidental and interval appendicectomies the charts of 1,014 patients who had had an appendicectomy for suspected appendicitis were reviewed. There were 986 cases of appendicitis and 28 negative appendicectomies (NAR: 2.76%). There were 741 adults and 273 paediatric patients (Table 1). Faecoliths were found in 18.1% (178/986) of positive appendicectomy specimens and in 28.6% (8/28) of negative appendicectomy specimens.

In the paediatric age group, the faecolith prevalence rate was 29.9% (79/264) in positive cases and 22.2% (2/9) in negative cases. Reducing the threshold age to ≤14 years did not affect prevalence rates (29.0%). In adults, faecolith prevalence was 13.7% (99/722) in positive cases and 31.6% (6/19) in negative appendicectomies. The difference between paediatric and adult groups was statistically significant (*p*<0.05).

In perforated appendicitis cases, the overall prevalence rate for faecoliths was 39.4% (54/137) compared with 14.6% (124/849) in non-perforated appendicitis (OR: 3.79, 95% confidence interval [CI]: 2.5–5.6, *p*<0.05). For adults, faecolith prevalence in perforation was 27.5% (22/80) compared with 12.0% (77/642) for non-perforated appendicitis (OR: 2.77, 95% CI: 1.6–4.7, *p*<0.05). For paediatric patients, faecolith prevalence in perforation was 56.1% (32/57) compared with 22.7% (47/207) for non-perforated appendicitis (OR: 4.35, 95% CI: 2.35–8.06, *p*=0.00). The difference in faecolith prevalence rates for non-perforated appendicitis between adults (12.0%) and paediatric patients (22.7%) was also statistically significant (OR: 2.15, 95% CI: 1.44–3.22, *p*=0.00).

Of the 1,014 patients, 888 had preoperative CT and 126 did not. Overall, 192 CT scans were read as showing faecoliths but only 86 were confirmed on pathology (PPV: 44.8%). Furthermore, 186 patients had faecoliths on pathology and 162 of these patients had CT but only 86 faecoliths were identified on CT (sensitivity 53.1%) (Table 2).

## Discussion

### Obstruction as the cause of appendicitis

Modern textbooks of surgery, internal medicine and pathology still teach that obstruction is the main cause of appendicitis and faecoliths are the main cause of obstruction in adults.^1–4^ This concept may have originated as early as 1846 with Volz,^7^ who observed a ‘principal pathogenetic agent’ for pericaecal inflammation (typhlitis) in faecoliths. In his seminal 1886 presentation, Fitz^5^ cited Matterstock’s finding of 53% faecal concretions in 169 fatal cases of appendicitis and his own finding of faecoliths in 47% of cases of perforating appendicitis as evidence implicating the faecolith as a main cause of appendicitis.

This belief gained momentum with the experiments and observations of van Zwalenberg,^8–10^ Wangensteen^11,12^ and Bowers.^13^ Wangensteen demonstrated that experimental obstruction of the appendix reproduces the inflammatory response and the clinical picture of appendicitis but the conclusion that obstruction is the main cause of clinical appendicitis is largely inferred. Bowers found faecoliths in 67% of 372 cases, concluding that obstruction by impacted faecoliths was the main cause of appendicitis.^13^ Support for the obstruction hypothesis has also come from more recent experimental work confirming the effects of obstruction on the appendix.^14^ Non-filling of the appendix on a barium enema and, more recently, on CT has been considered an indication for appendicectomy,^15,38^ based on the premise that obstruction of the lumen of the appendix is an important element of appendicitis.

**Table 2:** Faecoliths on computed tomography (CT) compared with faecoliths in pathology specimens

CT findings	Pathology positive for faecoliths	Pathology negative for faecoliths	Total
CT positive for faecolith	86	106	**192**
CT negative for faecolith	76	620	**696**
**Total**	**162**	**736**	**888**

However, which came first: the inflammation or the obstruction? The obstruction hypothesis assumes a cause of obstruction and theories on such causes abound. In the most popular description, a pre-existing intramural or extramural narrowing of the lumen of the appendix (stricture, neoplasm, lymphoid hyperplasia, Gerlach valve or adhesion) creates a partial obstruction, which is completed by an intraluminal entity. The most frequently mentioned entity has been the faecolith but parasites and foreign bodies have also been implicated. In several studies, faecoliths were also seen in significant numbers in the normal appendix, suggesting that they are often merely incidental findings.^16,17^ In children, lymphoid hyperplasia is accepted as the most frequent cause of obstruction although evidence for this is sparse.

The obstruction hypothesis has been challenged by some.^18–21^ Aschoff proposed that bacterial infection and not obstruction of the appendix was the inciting event.^18^ Arnbjörnsson’s experimental work also challenges the obstruction hypothesis.^19^ In an analysis of available data, Carr makes a case against obstruction as the main aetiology,^20^ suggesting several alternatives previously dismissed as unlikely by Bowers.^13^ These include infection, diet, trauma, genetics and even hypersensitivity.

### Faecoliths and appendicitis

Support for the theory that obstruction by faecoliths is the main cause of appendicitis comes from older studies (pre-1970) showing a high prevalence of faecoliths in appendicitis. In the largest study of appendix specimens to date, Collins described a faecolith prevalence of 44.25% in 71,000 specimens.^22^ These included 12,119 prophylactic appendicectomies and 6,409 postmortem specimens but only 11,961 cases of simple acute appendicitis. Since prevalence for each category is not identified, conclusions about an association with acute appendicitis cannot be made.

Several other studies have also demonstrated faecolith prevalence rates of 33–44% in appendicitis.^23–25^ However, recent large series (post-1970) demonstrate a low prevalence of faecoliths in appendicitis with rates ranging from 1.54% to 15%.^21,26–31^ A few small post-1970 studies (<100 cases) have described faecolith prevalence rates of 52% in appendicitis but 32–40% in incidental appendicectomies.^16,17^ The current study showed an overall faecolith prevalence rate of 18.1%, with 14.6% in non-perforated appendicitis and a rate of 39.4% in perforation.

Possible explanations for the higher prevalence of faecoliths in older studies include:

1. Patterns of appendicitis in the US are changing and faecoliths are less frequent now. Studies from Minnesota, where earlier faecolith prevalence rates of 39–44%^11,13,22^ decreased to 11% by 1990,^29^ support this view.
2. Different aetiologies apply in different regions of the country/world.
3. Older studies had much higher perforation rates and more severe forms of appendicitis, where faecoliths play a larger role.

In the modern era with low faecolith prevalence rates and low perforation rates, we submit that it is no longer valid to state that faecoliths are the main cause of appendicitis.

### Faecoliths in paediatric appendicitis

When only paediatric studies were considered, the prevalence of faecoliths in children (≤17 years) with appendicitis was consistently higher (19–65%)^32–34^ than in adults. Our study confirms this with a rate of 29.9% in children versus 13.7% in adults. The perforation rate for paediatric patients in our study was higher (21.6%) than in adults (11.1%) and faecolith prevalence in paediatric patients with perforation was 56.1% compared with 27.5% in adults. However, faecolith prevalence in non-perforated paediatric appendicitis was also significantly higher (22.7%) than in non-perforated adult appendicitis (12.0%). We conclude that faecoliths are more common in paediatric appendicitis than in adult appendicitis, independent of perforation.

### Faecoliths and perforation

Faecoliths were associated with perforation more often than with uncomplicated appendicitis in our study (39.4% vs 14.6%). This is similar to the findings of Fitz,^5^ Matterstock and Wangensteen.^11^ A classification of faecoliths into appendiceal calculi, calcified faecoliths and faecal pellets based on consistency and calcium content has been suggested by some authors who correlate calcified concretions with perforation.^30^ On the other hand, terms such as coprostasis and viscid faecal matter have been used to describe softer stool in the appendix.^31,35^ These authors suggest that these softer entities in the appendix are associated with appendicitis more often than the harder faecoliths. This militates against an obstructive aetiology. Others describe milking faecoliths intraoperatively from the appendix into the caecum,^16^ implying non-impaction and making obstruction less likely.

### Faecoliths on CT and in pathology specimens

Faecoliths first described on plain abdominal x-rays by Weisflog in 1906^36^ were probably appendiceal calculi or calcified faecoliths. Their presence on plain abdominal x-ray was considered a reliable sign of appendicitis, correlating well with faecoliths on pathology in 70%.^37^ On the other hand, CT is more sensitive, detecting even non-calcified faecoliths. Some authors have found good correlation (65–100%) between faecoliths on CT and appendicitis^32,38^ while others have not.^39^ They do not, however, correlate faecoliths on CT with faecoliths in the pathology specimen. Our study showed that CT has a sensitivity of just 53.1% and a PPV of 44.8% for identifying faecoliths in the appendicectomy specimen.

### Alternative causes of appendicitis

Other aetiologies have been suggested as the inciting event in appendicitis including catarrhal inflammation and lymphoid hyperplasia due to viral or bacterial infection, constipation, trauma, diet, genetic predilection, hypersensitivity and mucosal ulceration. Of these, catarrhal inflammation and constipation, both suggested a century ago,^18^ appear to be the most credible alternatives to obstruction.

Epidemiological studies suggest that constipation may be an important factor in the pathogenesis of appendicitis. According to studies from Africa and North America, populations on high fibre diets have a lower incidence of appendicitis than those on more westernised diets.^16,40,41^ Despite some experimental studies,^42,43^ real evidence of a causative relationship is lacking.

Obstruction probably plays a key role in the progression of appendicitis but evidence for faecoliths as the most common cause of uncomplicated appendicitis is weak. More than one mechanism appears to cause appendicitis, perhaps explaining why some cases of appendicitis seem to resolve without surgical intervention. We believe large scale, prospective studies are needed to re-examine the aetiopathology of a disease that is still the most frequent surgical emergency.

### Limitations

In a retrospective study, some faecoliths may have been lost before the specimen arrived in the pathology unit but it is most unlikely that this was a significant number. CT readings are interpreter/operator dependent but the data are presented without bias as officially interpreted.

## Conclusions

Based on the low prevalence of faecoliths in modern day appendicitis, the mantra that faecoliths are still the main cause of appendicitis is unsupported by the evidence. The role of the faecolith is unproven in non-perforated appendicitis. Contrary to popular belief, faecoliths are more common in paediatric appendicitis than in adult appendicitis. CT is a poor predictor of faecoliths in the appendicectomy specimen.
